# *rac*-3,9-Bis(3-chloro­phen­yl)-2,4,8,10-tetra­oxaspiro­[5.5]undeca­ne

**DOI:** 10.1107/S1600536811037172

**Published:** 2011-09-17

**Authors:** Zhengyi Li, Beibei Zhou, Liang Chen, Ling Ge, Xiaoqiang Sun

**Affiliations:** a Key Laboratory of Fine Chemical Engineering, Changzhou University, Changzhou 213164, Jiangsu, People’s Republic of China

## Abstract

In the title compound, C_19_H_18_Cl_2_O_4_, the two non-planar six-membered heterocycles passing through the spiro-C atom both adopt chair conformations, and the dihedral angle between the two benzene rings is 7.2 (1)°. In the crystal, the enanti­omers with *R* and *S* configurations are generated by the symmetry elements of the centrosymmetric space group, forming a racemic crystal. Inter­molecular C—H⋯π and weak C—H⋯O inter­actions link the mol­ecules in the crystal structure.

## Related literature

For general background to spiranes, see: Cismaş *et al.* (2005 ▶); Mihiş *et al.* (2008 ▶); Sun *et al.* (2010 ▶).
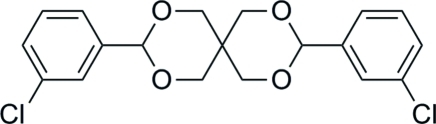


## Experimental

### 

#### Crystal data

C_19_H_18_Cl_2_O_4_
*M* *_r_* = 381.23
Monoclinic, 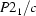

*a* = 13.0924 (13) Å
*b* = 5.8473 (6) Å
*c* = 23.061 (2) Å
β = 92.865 (2)°
*V* = 1763.2 (3) Å^3^
*Z* = 4
Mo *K*α radiation
μ = 0.39 mm^−1^
*T* = 296 K
0.30 × 0.20 × 0.20 mm

#### Data collection

Bruker APEXII CCD diffractometer
Absorption correction: multi-scan (*SADABS*; Bruker, 2000 ▶) *T*_min_ = 0.892, *T*_max_ = 0.926
9216 measured reflections
3083 independent reflections
2669 reflections with *I* > 2σ(*I*)
*R*_int_ = 0.044

#### Refinement

*R*[*F* ^2^ > 2σ(*F* ^2^)] = 0.034
*wR*(*F* ^2^) = 0.091
*S* = 1.01
3083 reflections
227 parameters
H-atom parameters constrained
Δρ_max_ = 0.23 e Å^−3^
Δρ_min_ = −0.37 e Å^−3^

### 

Data collection: *APEX2* (Bruker, 2000 ▶); cell refinement: *SAINT* (Bruker, 2000 ▶); data reduction: *SAINT*; program(s) used to solve structure: *SHELXTL* (Sheldrick, 2008 ▶); program(s) used to refine structure: *SHELXTL*; molecular graphics: *SHELXTL*; software used to prepare material for publication: *SHELXTL*.

## Supplementary Material

Crystal structure: contains datablock(s) I, global. DOI: 10.1107/S1600536811037172/kp2345sup1.cif

Structure factors: contains datablock(s) I. DOI: 10.1107/S1600536811037172/kp2345Isup2.hkl

Supplementary material file. DOI: 10.1107/S1600536811037172/kp2345Isup3.cml

Additional supplementary materials:  crystallographic information; 3D view; checkCIF report

## Figures and Tables

**Table 1:** Hydrogen-bond geometry (Å, °) *Cg*1 is the centroid of the C1–C6 ring.

*D*—H⋯*A*	*D*—H	H⋯*A*	*D*⋯*A*	*D*—H⋯*A*
C12—H12*B*⋯*Cg*1^i^	0.97	2.70	3.632 (2)	162
C9—H9*A*⋯O3^ii^	0.97	2.64	3.402 (2)	135
C11—H11*B*⋯O3^iii^	0.97	2.61	3.530 (2)	158

Symmetry codes: (i) 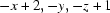; (ii) 
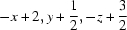
; (iii) 
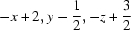
.

**Fig. 1.**
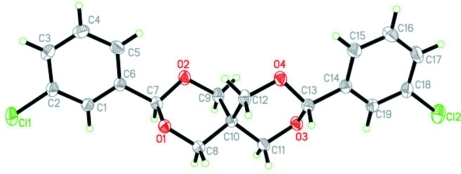
The molecular structure of the title compound. Displacement ellipsoids are drawn at the 30% probability level.

**Fig. 2.**
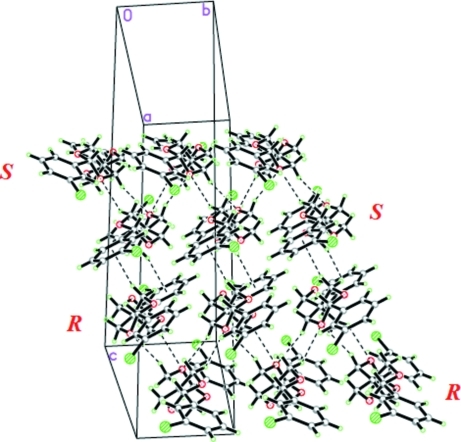
Crystal packing of (I). Hydrogen bonds are shown as dashed lines.

## supplementary crystallographic information

### Comment

Owing to the characteristic axial and helical chirality, the stereochemistry of spiranes with six-membered rings has been extensively studied (Cismaş *et al.*, 2005). In the past three decades, most of these investigations were carried out with spiranes containing 1,3-dioxane units (Mihiş *et al.*, 2008; Sun *et al.*, 2010). We herein present the structure of 3,9-bis(3-chlorophenyl)-2,4,8,10-tetraoxaspiro[5.5]undecane (Fig. 1).

In the title compound, the two nonplanar six-membered heterocycles [(O1, O2 and C7–C10) and (O3, O4 and C10–C13)] passing through the spiro-C atom (C10) both adopt chair conformations, and the dihedral angle between the two benzene rings (C1–C6 and C14–C19) is 7.2 (1)°. In the crystal packing structure (Fig. 2), the enantiomers with *R* and *S* configurations are generated by the symmetry elements of the centrosymmetric groups forming a racemate. Intermolecular weak C–H···O interactions link molecules with the same configuration (*R* or *S*) into the two chains chains along the *b* axis. The chains are further connected by C–H···π interactions [C12–H12B···*Cg*1^i^ = 2.70 Å; (i) -*x* + 2, -*y*, -*z* + 1; *Cg*1 is the centroid of benzene ring (C1–C6); Table 1] creating a racemic network.

### Experimental

To a solution of 3-chlorobenzaldehyde (7.32 mmol, 1.03 g) and pentaerythritol (4 mmol, 0.54 g) in toluene (30 mL), phosphotungstic acid (30 mg) as catalyst was added, respectively. The mixtures were refluxed for 4 h to complete the reaction. After reaction, the mixture was evaporated under vacuum and the residuces were washed with 5% sodium bicarboinate (20 mL) and 50% ethanol (20 mL), respectively. The pure product recrystallised from ethanol to afford colourless crystals (yield 65%, m.p. 397–398 K). Single crystals suitable for X-ray diffraction were also obtained by evaporation of an enthanol solution.

### Refinement

All the H atoms were placed in geometrically idealized positions and constrained to ride on their parent atoms, with C—H distances of 0.93–0.98 Å, and with *U*_iso_(H) = 1.2*U*_eq_(C).

### Crystal data

**Table:**

C_19_H_18_Cl_2_O_4_	*F*(000) = 792
*M**_r_* = 381.23	*D*_x_ = 1.436 Mg m^−^^3^
Monoclinic, *P*2_1_/*c*	Melting point: 397 K
Hall symbol: -P 2ybc	Mo *K*α radiation, λ = 0.71073 Å
*a* = 13.0924 (13) Å	Cell parameters from 5568 reflections
*b* = 5.8473 (6) Å	θ = 2.4–30.2°
*c* = 23.061 (2) Å	µ = 0.39 mm^−^^1^
β = 92.865 (2)°	*T* = 296 K
*V* = 1763.2 (3) Å^3^	PRISM, colourless
*Z* = 4	0.30 × 0.20 × 0.20 mm

### Data collection

**Table:**

Bruker APEXII CCD diffractometer	3083 independent reflections
Radiation source: fine-focus sealed tube	2669 reflections with *I* > 2σ(*I*)
graphite	*R*_int_ = 0.044
φ and ω scans	θ_max_ = 25.0°, θ_min_ = 1.6°
Absorption correction: multi-scan (*SADABS*; Bruker, 2000)	*h* = −15→15
*T*_min_ = 0.892, *T*_max_ = 0.926	*k* = −6→6
9216 measured reflections	*l* = −27→23

### Refinement

**Table:**

Refinement on *F*^2^	Secondary atom site location: difference Fourier map
Least-squares matrix: full	Hydrogen site location: inferred from neighbouring sites
*R*[*F*^2^ > 2σ(*F*^2^)] = 0.034	H-atom parameters constrained
*wR*(*F*^2^) = 0.091	*w* = 1/[σ^2^(*F*_o_^2^) + (0.045*P*)^2^ + 0.585*P*] where *P* = (*F*_o_^2^ + 2*F*_c_^2^)/3
*S* = 1.01	(Δ/σ)_max_ = 0.001
3083 reflections	Δρ_max_ = 0.23 e Å^−^^3^
227 parameters	Δρ_min_ = −0.37 e Å^−^^3^
0 restraints	Extinction correction: *SHELXTL* (Sheldrick, 2008), Fc^*^=kFc[1+0.001xFc^2^λ^3^/sin(2θ)]^-1/4^
Primary atom site location: structure-invariant direct methods	Extinction coefficient: 0.0101 (15)

### Special details

**Table:**

Geometry. All e.s.d.'s (except the e.s.d. in the dihedral angle between two l.s. planes) are estimated using the full covariance matrix. The cell e.s.d.'s are taken into account individually in the estimation of e.s.d.'s in distances, angles and torsion angles; correlations between e.s.d.'s in cell parameters are only used when they are defined by crystal symmetry. An approximate (isotropic) treatment of cell e.s.d.'s is used for estimating e.s.d.'s involving l.s. planes.
Refinement. Refinement of *F*^2^ against ALL reflections. The weighted *R*-factor *wR* and goodness of fit *S* are based on *F*^2^, conventional *R*-factors *R* are based on *F*, with *F* set to zero for negative *F*^2^. The threshold expression of *F*^2^ > σ(*F*^2^) is used only for calculating *R*-factors(gt) *etc*. and is not relevant to the choice of reflections for refinement. *R*-factors based on *F*^2^ are statistically about twice as large as those based on *F*, and *R*- factors based on ALL data will be even larger.

### Fractional atomic coordinates and isotropic or equivalent isotropic displacement parameters (Å^2^)

**Table:**

	*x*	*y*	*z*	*U*_iso_*/*U*_eq_	
Cl1	1.44666 (4)	−0.09142 (9)	0.42374 (2)	0.06281 (18)	
Cl2	0.60790 (4)	0.03575 (11)	0.87193 (2)	0.0707 (2)	
C10	1.00108 (11)	−0.0663 (3)	0.63687 (6)	0.0355 (3)	
C7	1.18050 (11)	0.0270 (3)	0.57866 (6)	0.0381 (4)	
H7	1.2282	−0.0646	0.6032	0.046*	
C13	0.81522 (11)	0.0410 (3)	0.68608 (6)	0.0367 (3)	
H13	0.7765	−0.0790	0.6650	0.044*	
C14	0.74431 (11)	0.1662 (3)	0.72470 (6)	0.0365 (3)	
C6	1.23665 (11)	0.1316 (3)	0.52994 (6)	0.0360 (3)	
C9	1.08461 (12)	0.0975 (3)	0.65948 (7)	0.0437 (4)	
H9A	1.0544	0.2177	0.6820	0.052*	
H9B	1.1333	0.0154	0.6848	0.052*	
C12	0.91461 (12)	0.0658 (3)	0.60524 (6)	0.0426 (4)	
H12A	0.9431	0.1715	0.5779	0.051*	
H12B	0.8704	−0.0401	0.5835	0.051*	
C19	0.71569 (12)	0.0629 (3)	0.77554 (7)	0.0407 (4)	
H19	0.7439	−0.0767	0.7871	0.049*	
C18	0.64487 (12)	0.1693 (3)	0.80883 (7)	0.0452 (4)	
C1	1.31014 (11)	−0.0007 (3)	0.50473 (7)	0.0383 (3)	
H1	1.3269	−0.1442	0.5198	0.046*	
C11	0.95635 (13)	−0.2022 (3)	0.68599 (7)	0.0466 (4)	
H11A	0.9156	−0.3274	0.6697	0.056*	
H11B	1.0116	−0.2670	0.7103	0.056*	
C15	0.70256 (13)	0.3758 (3)	0.70861 (8)	0.0475 (4)	
H15	0.7217	0.4465	0.6747	0.057*	
C2	1.35834 (12)	0.0819 (3)	0.45714 (7)	0.0413 (4)	
C8	1.04953 (14)	−0.2341 (3)	0.59572 (8)	0.0479 (4)	
H8A	1.0958	−0.3346	0.6178	0.057*	
H8B	0.9965	−0.3276	0.5768	0.057*	
C3	1.33736 (13)	0.2967 (3)	0.43493 (7)	0.0471 (4)	
H3	1.3716	0.3520	0.4035	0.057*	
C5	1.21370 (13)	0.3469 (3)	0.50771 (7)	0.0451 (4)	
H5	1.1645	0.4368	0.5244	0.054*	
C17	0.60229 (13)	0.3780 (3)	0.79319 (8)	0.0533 (5)	
H17	0.5546	0.4477	0.8160	0.064*	
C4	1.26424 (14)	0.4277 (3)	0.46054 (8)	0.0505 (4)	
H4	1.2487	0.5724	0.4459	0.061*	
C16	0.63242 (14)	0.4803 (3)	0.74285 (9)	0.0567 (5)	
H16	0.6052	0.6216	0.7318	0.068*	
O3	0.89369 (8)	−0.06191 (19)	0.72093 (4)	0.0415 (3)	
O1	1.10447 (8)	−0.11743 (19)	0.55266 (5)	0.0421 (3)	
O4	0.85569 (8)	0.1908 (2)	0.64567 (4)	0.0429 (3)	
O2	1.13674 (8)	0.19688 (19)	0.61245 (5)	0.0441 (3)	

### Atomic displacement parameters (Å^2^)

**Table:**

	*U*^11^	*U*^22^	*U*^33^	*U*^12^	*U*^13^	*U*^23^
Cl1	0.0534 (3)	0.0747 (4)	0.0629 (3)	0.0064 (2)	0.0281 (2)	−0.0002 (2)
Cl2	0.0728 (3)	0.0869 (4)	0.0552 (3)	−0.0017 (3)	0.0312 (2)	0.0047 (3)
C10	0.0400 (8)	0.0339 (8)	0.0332 (7)	0.0029 (6)	0.0077 (6)	−0.0012 (6)
C7	0.0375 (8)	0.0435 (9)	0.0333 (8)	0.0046 (7)	0.0032 (6)	−0.0027 (6)
C13	0.0374 (8)	0.0396 (8)	0.0333 (8)	−0.0012 (6)	0.0030 (6)	−0.0001 (6)
C14	0.0320 (7)	0.0410 (8)	0.0365 (8)	−0.0008 (6)	0.0015 (6)	−0.0031 (6)
C6	0.0364 (8)	0.0403 (8)	0.0314 (7)	−0.0037 (6)	0.0015 (6)	−0.0043 (6)
C9	0.0440 (8)	0.0575 (10)	0.0301 (8)	−0.0008 (8)	0.0061 (6)	−0.0084 (7)
C12	0.0412 (8)	0.0578 (10)	0.0293 (7)	0.0037 (7)	0.0056 (6)	0.0027 (7)
C19	0.0395 (8)	0.0414 (9)	0.0415 (8)	−0.0005 (7)	0.0050 (7)	−0.0017 (7)
C18	0.0392 (8)	0.0554 (10)	0.0417 (9)	−0.0061 (8)	0.0099 (7)	−0.0063 (7)
C1	0.0373 (8)	0.0393 (8)	0.0384 (8)	−0.0006 (7)	0.0029 (6)	0.0014 (6)
C11	0.0546 (10)	0.0402 (9)	0.0467 (9)	0.0128 (8)	0.0180 (7)	0.0077 (7)
C15	0.0473 (9)	0.0479 (10)	0.0478 (9)	0.0060 (8)	0.0067 (7)	0.0053 (8)
C2	0.0353 (8)	0.0496 (9)	0.0394 (8)	−0.0052 (7)	0.0057 (6)	−0.0027 (7)
C8	0.0592 (10)	0.0354 (9)	0.0511 (9)	−0.0028 (8)	0.0229 (8)	−0.0051 (7)
C3	0.0516 (9)	0.0505 (10)	0.0395 (8)	−0.0119 (8)	0.0049 (7)	0.0046 (7)
C5	0.0533 (9)	0.0393 (9)	0.0426 (9)	0.0041 (7)	0.0032 (7)	−0.0061 (7)
C17	0.0423 (9)	0.0600 (11)	0.0587 (11)	0.0058 (8)	0.0130 (8)	−0.0143 (9)
C4	0.0662 (11)	0.0379 (9)	0.0471 (9)	−0.0035 (8)	0.0004 (8)	0.0042 (7)
C16	0.0528 (10)	0.0491 (10)	0.0686 (12)	0.0148 (9)	0.0065 (9)	−0.0016 (9)
O3	0.0461 (6)	0.0455 (6)	0.0338 (5)	0.0124 (5)	0.0106 (5)	0.0071 (5)
O1	0.0510 (6)	0.0384 (6)	0.0382 (6)	−0.0059 (5)	0.0149 (5)	−0.0094 (5)
O4	0.0453 (6)	0.0475 (6)	0.0369 (6)	0.0117 (5)	0.0112 (5)	0.0103 (5)
O2	0.0475 (6)	0.0464 (6)	0.0395 (6)	−0.0082 (5)	0.0139 (5)	−0.0138 (5)

### Geometric parameters (Å, °)

**Table:**

Cl1—C2	1.7456 (16)	C12—H12B	0.9700
Cl2—C18	1.7413 (17)	C19—C18	1.381 (2)
C10—C11	1.524 (2)	C19—H19	0.9300
C10—C8	1.525 (2)	C18—C17	1.382 (3)
C10—C9	1.526 (2)	C1—C2	1.380 (2)
C10—C12	1.526 (2)	C1—H1	0.9300
C7—O2	1.4026 (18)	C11—O3	1.4356 (18)
C7—O1	1.4153 (19)	C11—H11A	0.9700
C7—C6	1.503 (2)	C11—H11B	0.9700
C7—H7	0.9800	C15—C16	1.383 (2)
C13—O4	1.4019 (18)	C15—H15	0.9300
C13—O3	1.4074 (18)	C2—C3	1.379 (2)
C13—C14	1.508 (2)	C8—O1	1.4285 (19)
C13—H13	0.9800	C8—H8A	0.9700
C14—C15	1.385 (2)	C8—H8B	0.9700
C14—C19	1.387 (2)	C3—C4	1.382 (2)
C6—C1	1.385 (2)	C3—H3	0.9300
C6—C5	1.387 (2)	C5—C4	1.384 (2)
C9—O2	1.4332 (19)	C5—H5	0.9300
C9—H9A	0.9700	C17—C16	1.381 (3)
C9—H9B	0.9700	C17—H17	0.9300
C12—O4	1.4388 (18)	C4—H4	0.9300
C12—H12A	0.9700	C16—H16	0.9300
			
C11—C10—C8	108.33 (13)	C17—C18—Cl2	119.26 (13)
C11—C10—C9	111.63 (14)	C2—C1—C6	119.55 (15)
C8—C10—C9	107.65 (13)	C2—C1—H1	120.2
C11—C10—C12	108.41 (13)	C6—C1—H1	120.2
C8—C10—C12	110.49 (13)	O3—C11—C10	111.77 (12)
C9—C10—C12	110.32 (14)	O3—C11—H11A	109.3
O2—C7—O1	111.08 (12)	C10—C11—H11A	109.3
O2—C7—C6	110.82 (13)	O3—C11—H11B	109.3
O1—C7—C6	106.63 (12)	C10—C11—H11B	109.3
O2—C7—H7	109.4	H11A—C11—H11B	107.9
O1—C7—H7	109.4	C16—C15—C14	120.17 (16)
C6—C7—H7	109.4	C16—C15—H15	119.9
O4—C13—O3	110.93 (12)	C14—C15—H15	119.9
O4—C13—C14	110.70 (12)	C3—C2—C1	121.58 (15)
O3—C13—C14	108.89 (12)	C3—C2—Cl1	119.28 (12)
O4—C13—H13	108.8	C1—C2—Cl1	119.14 (13)
O3—C13—H13	108.8	O1—C8—C10	111.40 (13)
C14—C13—H13	108.8	O1—C8—H8A	109.3
C15—C14—C19	119.48 (15)	C10—C8—H8A	109.3
C15—C14—C13	121.20 (14)	O1—C8—H8B	109.3
C19—C14—C13	119.21 (14)	C10—C8—H8B	109.3
C1—C6—C5	119.62 (14)	H8A—C8—H8B	108.0
C1—C6—C7	117.54 (14)	C2—C3—C4	118.41 (15)
C5—C6—C7	122.74 (14)	C2—C3—H3	120.8
O2—C9—C10	110.88 (12)	C4—C3—H3	120.8
O2—C9—H9A	109.5	C4—C5—C6	119.81 (16)
C10—C9—H9A	109.5	C4—C5—H5	120.1
O2—C9—H9B	109.5	C6—C5—H5	120.1
C10—C9—H9B	109.5	C16—C17—C18	118.26 (16)
H9A—C9—H9B	108.1	C16—C17—H17	120.9
O4—C12—C10	110.87 (12)	C18—C17—H17	120.9
O4—C12—H12A	109.5	C3—C4—C5	121.02 (16)
C10—C12—H12A	109.5	C3—C4—H4	119.5
O4—C12—H12B	109.5	C5—C4—H4	119.5
C10—C12—H12B	109.5	C17—C16—C15	120.94 (17)
H12A—C12—H12B	108.1	C17—C16—H16	119.5
C18—C19—C14	119.40 (15)	C15—C16—H16	119.5
C18—C19—H19	120.3	C13—O3—C11	110.19 (12)
C14—C19—H19	120.3	C7—O1—C8	111.00 (12)
C19—C18—C17	121.73 (16)	C13—O4—C12	110.26 (12)
C19—C18—Cl2	119.00 (14)	C7—O2—C9	110.87 (12)
			
O4—C13—C14—C15	19.0 (2)	C6—C1—C2—Cl1	−177.16 (11)
O3—C13—C14—C15	141.19 (15)	C11—C10—C8—O1	−172.53 (14)
O4—C13—C14—C19	−164.95 (13)	C9—C10—C8—O1	−51.68 (18)
O3—C13—C14—C19	−42.74 (18)	C12—C10—C8—O1	68.85 (18)
O2—C7—C6—C1	159.48 (13)	C1—C2—C3—C4	−1.7 (2)
O1—C7—C6—C1	−79.51 (16)	Cl1—C2—C3—C4	177.45 (13)
O2—C7—C6—C5	−24.2 (2)	C1—C6—C5—C4	0.1 (2)
O1—C7—C6—C5	96.80 (17)	C7—C6—C5—C4	−176.16 (15)
C11—C10—C9—O2	170.89 (12)	C19—C18—C17—C16	−0.1 (3)
C8—C10—C9—O2	52.13 (17)	Cl2—C18—C17—C16	−179.73 (15)
C12—C10—C9—O2	−68.50 (16)	C2—C3—C4—C5	0.6 (3)
C11—C10—C12—O4	50.16 (18)	C6—C5—C4—C3	0.2 (3)
C8—C10—C12—O4	168.73 (13)	C18—C17—C16—C15	0.7 (3)
C9—C10—C12—O4	−72.36 (16)	C14—C15—C16—C17	−0.4 (3)
C15—C14—C19—C18	0.9 (2)	O4—C13—O3—C11	−63.94 (16)
C13—C14—C19—C18	−175.20 (14)	C14—C13—O3—C11	173.99 (12)
C14—C19—C18—C17	−0.7 (2)	C10—C11—O3—C13	56.69 (18)
C14—C19—C18—Cl2	178.92 (12)	O2—C7—O1—C8	−61.88 (16)
C5—C6—C1—C2	−1.1 (2)	C6—C7—O1—C8	177.28 (12)
C7—C6—C1—C2	175.31 (14)	C10—C8—O1—C7	57.07 (18)
C8—C10—C11—O3	−169.67 (13)	O3—C13—O4—C12	64.98 (15)
C9—C10—C11—O3	71.97 (18)	C14—C13—O4—C12	−174.01 (11)
C12—C10—C11—O3	−49.75 (18)	C10—C12—O4—C13	−58.23 (16)
C19—C14—C15—C16	−0.4 (3)	O1—C7—O2—C9	62.62 (16)
C13—C14—C15—C16	175.67 (16)	C6—C7—O2—C9	−179.05 (12)
C6—C1—C2—C3	2.0 (2)	C10—C9—O2—C7	−58.53 (16)

### Hydrogen-bond geometry (Å, °)

**Table:**

Cg1 is the centroid of the C1–C6 ring.

**Table:**

*D*—H···*A*	*D*—H	H···*A*	*D*···*A*	*D*—H···*A*
C12—H12B···Cg1^i^	0.97	2.70	3.632 (2)	162.
C9—H9A···O3^ii^	0.97	2.64	3.402 (2)	135.
C11—H11B···O3^iii^	0.97	2.61	3.530 (2)	158.

Symmetry codes: (i) −*x*+2, −*y*, −*z*+1; (ii) −*x*+2, *y*+1/2, −*z*+3/2; (iii) −*x*+2, *y*−1/2, −*z*+3/2.
